# Workplace Violence Against Physicians in Bangladesh: A Contemporary Systems‐Based Narrative Review of Current Evidence

**DOI:** 10.1002/puh2.70452

**Published:** 2026-11-07

**Authors:** Md. Abu Sufian, Nawshin Sayara Satter, Md. Monir Hossain Shimul

**Affiliations:** ^1^ Bangladesh University of Health Sciences Dhaka Bangladesh; ^2^ International Centre for Diarrhoeal Disease Research Bangladesh Dhaka Bangladesh; ^3^ Department of Public Health & Informatics Bangladesh University of Professionals Dhaka Bangladesh; ^4^ Continental Hospital PLC Dhaka Bangladesh; ^5^ Department of Public Health Daffodil International University Dhaka Bangladesh; ^6^ Faculty of Health Education and Life Sciences Birmingham City University Birmingham UK

**Keywords:** healthcare workers, occupational health, physicians, psychological violence, workplace violence

## Abstract

**Background:**

Workplace violence against physicians is an occupational and public health challenge in resource‐constrained healthcare systems. In Bangladesh, physician shortages, overcrowding, service delays, inadequate security, and institutional constraints may increase exposure to clinical environments. This narrative review synthesized evidence on forms, perpetrators, determinants, consequences, institutional responses, and prevention using a systems‐based and socio‐ecological perspective.

**Methods:**

A narrative review was conducted with reference to the Scale for the Assessment of Narrative Review Articles (SANRA). PubMed, Scopus, Web of Science, and Google Scholar were searched for peer‐reviewed publications from January 2022 to May 2026. The primary evidence comprised studies reporting workplace violence among physicians or physician‐specific findings in Bangladeshi healthcare settings. Contextual evidence was considered separately to inform interpretation. Methodological quality was assessed using Joanna Briggs Institute critical appraisal tools. Findings were synthesized thematically because methodological heterogeneity precluded quantitative pooling.

**Results:**

Twelve studies constituted the evidence base. Psychological and verbal violence were most common, with patients’ relatives or attendants frequently identified as perpetrators. Younger and early‐career physicians, emergency department staff, and night‐shift workers appeared particularly vulnerable, although demographic associations varied. Recurring determinants included treatment delays, overcrowding, staffing constraints, communication difficulties, inadequate security, weak reporting mechanisms, governance limitations, and health‐system pressures. Workplace violence was associated with psychological distress, burnout, reduced job satisfaction, defensive medical practice, workforce instability, and disruption of healthcare delivery. Institutional reporting, investigation, and post‐incident support remained inadequate.

**Conclusion:**

Workplace violence against physicians in Bangladesh is a systems‐level occupational, governance, and healthcare‐resilience challenge arising from interacting individual, interpersonal, organizational, health‐system, and societal conditions. Prevention should extend beyond reactive security measures to workforce strengthening, standardized reporting and accountability, legal protection, occupational support, communication and de‐escalation, and public engagement. Future research should prioritize standardized measurement, longitudinal and intervention studies, and underrepresented healthcare settings.

## Graphical abstract

Workplace violence against physicians in Bangladesh is a systems‐level challenge shaped by interacting individual, interpersonal, organizational, health‐system, and societal factors. Evidence from 12 studies indicates frequent verbal and psychological violence, particularly involving patients’ relatives or attendants, with important consequences for physician well‐being and healthcare delivery. Prevention requires coordinated action through workforce strengthening, standardized reporting and accountability, legal protection, occupational support, communication and de‐escalation training, and public engagement.

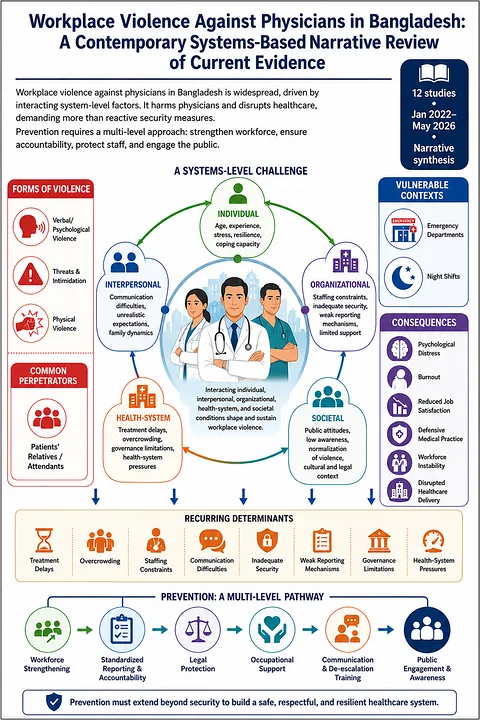

## 1 Introduction

Workplace violence against healthcare workers has become a major occupational and public health concern worldwide, particularly among physicians working in high‐pressure and resource‐constrained clinical environments [1]. The World Health Organization (WHO) defines workplace violence as incidents involving abuse, threats, harassment, or assaults related to work that may compromise the safety, well‐being, or health of healthcare workers, including physical, verbal, psychological, and sexual violence [2]. Violence against physicians is increasingly recognized as a critical challenge to healthcare delivery because it adversely affects psychological well‐being, professional performance, job satisfaction, workforce retention, and the quality of patient care [3, 4]. Workplace violence is increasingly understood as a multifactorial phenomenon arising from interactions among individual, interpersonal, organizational, health‐system, and societal factors. This systems perspective provides a useful framework for understanding workplace violence beyond isolated interpersonal conflicts.

The social ecological model provides the conceptual basis for interpreting the evidence synthesized in this review. Within this framework, workplace violence is understood not as an isolated interpersonal event but as an outcome of interacting influences operating at individual, interpersonal, organizational, health‐system, and societal levels. Individual and occupational characteristics may modify exposure, whereas interpersonal conflict occurs within organizational environments shaped by workload, staffing, waiting times, communication, security, and institutional support. These organizational conditions are themselves influenced by broader health‐system capacity, governance, socioeconomic conditions, and sociocultural expectations. International evidence further indicates that healthcare workers in low‐ and middle‐income countries are disproportionately exposed to workplace violence because of fragile health systems, workforce shortages, inadequate institutional security, overcrowded facilities, ineffective communication, and limited healthcare resources [3, 4].

Globally, workplace violence has been reported across emergency departments, intensive care units, outpatient clinics, and primary healthcare facilities, with psychological violence accounting for approximately 60%–90% of reported incidents, substantially exceeding physical violence [4, 5]. Similar patterns have been observed across South Asian countries, including India, Pakistan, China, and Nepal, where violence against physicians has emerged as a major challenge associated with overcrowded hospitals, prolonged waiting times, inadequate staffing, weak legal protection, and declining public trust in healthcare services [6, 7]. Patient relatives and attendants are frequently involved in violent incidents, particularly during emotionally stressful situations, such as treatment delays, critical illness, or death notifications [6, 8]. In addition, media reporting of adverse clinical outcomes and unrealistic public expectations of healthcare services may further intensify conflicts between healthcare providers and patients in resource‐constrained settings [9, 10].

Against this global backdrop, Bangladesh faces unique structural and organizational challenges that may further increase physicians’ vulnerability to workplace violence. As a densely populated lower‐middle‐income country with more than 170 million people, Bangladesh continues to experience substantial shortages of physicians, with approximately 0.6 physicians per 1000 population, well below recommended international standards [11, 12]. These workforce shortages, together with overcrowded public hospitals, limited infrastructure, shortages of medical supplies, inadequate security arrangements, and heavy clinical workloads, contribute to prolonged waiting times, rushed consultations, and impaired patient–provider communication [11, 12]. Consequently, physicians working in emergency departments, intensive care units, surgical services, and night shifts are particularly vulnerable because they routinely manage critically ill patients under conditions of high workload, emotional stress, and limited institutional support [13, 14].

Recent studies conducted in Bangladesh have consistently reported workplace violence ranging from verbal abuse and threats to severe physical assault [13, 14, 15, 16, 17]. Such incidents have been associated with psychological trauma, anxiety, burnout, fear, defensive medical practice, reduced work performance, absenteeism, and intentions to leave clinical practice [4, 13]. Beyond their effects on individual physicians, these incidents may compromise healthcare quality, disrupt continuity of care, weaken workforce retention, and reduce public confidence in the healthcare system.

Although several international reviews have examined workplace violence among healthcare professionals, they primarily synthesized evidence across multiple countries and professional groups without providing a comprehensive assessment of physicians in Bangladesh [3, 4]. Existing Bangladeshi studies remain fragmented, vary substantially in methodology, healthcare settings, outcome definitions, and measurement approaches, and have not previously been synthesized to identify consistent patterns, contextual determinants, institutional responses, and policy implications. The principal contribution of this review is therefore not simply to summarize reported prevalence but to integrate available Bangladeshi evidence across multiple levels of influence and to identify how physician vulnerability, interpersonal encounters, organizational conditions, and broader health‐system constraints interact in the emergence and persistence of workplace violence. The synthesis suggests that workplace violence in Bangladesh cannot be adequately explained by individual aggression or isolated patient–physician conflict. Instead, interpersonal violence may represent the immediate manifestation of deeper organizational and health‐system pressures, including overcrowding, treatment delays, workforce shortages, inadequate security, communication failures, sociocultural expectations, and governance weaknesses.

Accordingly, this narrative review synthesizes the available evidence on workplace violence against physicians in Bangladesh. Specifically, it examines the epidemiology and patterns of workplace violence, identifies associated risk factors and perpetrators, explores organizational and health‐system determinants, evaluates the consequences for physicians and healthcare delivery, reviews institutional responses and prevention strategies, and identifies important knowledge gaps to inform future research, policy, and practice. By integrating these dimensions within a socio‐ecological framework, this review advances the literature from a predominantly descriptive account of workplace violence toward a systems‐oriented interpretation of how violence emerges, affects physicians and healthcare delivery, and may be prevented.

## 2 Methodology

### 2.1 Study Design and Review Framework

A narrative review was conducted to synthesize the available evidence on workplace violence against physicians in Bangladesh. This approach was considered appropriate because the available literature was methodologically heterogeneous with respect to study designs, participant populations, healthcare settings, outcome definitions, and measurement approaches, making quantitative pooling inappropriate for the primary objectives of this review, while allowing a broader contextual understanding of the evidence. The review synthesized evidence on the epidemiology, types of workplace violence, associated risk factors, perpetrators, organizational and health‐system determinants, consequences, institutional responses, and prevention strategies relevant to physicians in Bangladesh.

The review protocol was developed before literature searching to define the review objectives, eligibility criteria, search strategy, study selection procedures, data extraction methods, quality appraisal, and evidence synthesis approach. Although a protocol was developed before literature searching, it was not prospectively registered in PROSPERO or another public review registry. This is acknowledged as a limitation of the review. The review was prepared in accordance with the Scale for the Assessment of Narrative Review Articles (SANRA) recommendations to enhance methodological transparency and reporting quality [3, 10].

### 2.2 Search Strategy

A comprehensive literature search was conducted in PubMed, Scopus, Web of Science, and Google Scholar. Additional records were identified through manual screening of reference lists, forward and backward citation tracking, and relevant regional sources to maximize retrieval of eligible studies. Searches were performed between March 25 and May 15, 2026 and covered publications from January 2022 to May 2026. This publication window was selected a priori to prioritize contemporary evidence reflecting the current organizational, workforce, governance, and healthcare‐delivery context in Bangladesh. Earlier publications were retained only as contextual or comparative evidence when they were necessary to interpret determinants, mechanisms, consequences, or prevention strategies.

The search strategy combined Medical Subject Headings (MeSH), controlled vocabulary where applicable, and free‐text terms related to workplace violence and physicians. An example search strategy was as follows: (“workplace violence” OR “occupational violence” OR assault OR aggression OR bullying) AND (physician* OR doctor* OR “medical practitioner*” OR “healthcare worker*”) AND (Bangladesh OR Bangladeshi). Database‐specific search syntax was adapted according to indexing systems, with searches performed within title, abstract, and keyword fields. Complete database‐specific search strategies, filters, and retrieved records are provided in Table S1.

For Google Scholar, only the first several hundred relevance‐ranked records were screened because subsequent results became progressively less relevant. Gray literature, including government reports, professional society publications, and organizational reports, was searched to identify potentially relevant evidence but was not formally included unless it satisfied the predefined eligibility criteria.

Only English‐language publications were included because peer‐reviewed scientific literature from Bangladesh is predominantly published in English and translation resources were unavailable. This restriction may have introduced language bias.

### 2.3 Eligibility Criteria

The primary evidence base comprised peer‐reviewed publications reporting workplace violence among physicians or providing extractable physician‐specific findings in Bangladeshi healthcare settings. Eligible publications were those published in English between January 2022 and May 2026. To provide a comprehensive narrative synthesis, the review included original quantitative and qualitative studies, content analyses, systematic reviews, narrative reviews, and other peer‐reviewed publications that contributed direct evidence on workplace violence among physicians or relevant contextual evidence informing the interpretation of its determinants, consequences, mechanisms, institutional responses, or prevention strategies. Earlier Bangladeshi publications and international evidence were considered separately as contextual evidence when they informed interpretation of determinants, consequences, mechanisms, or prevention strategies relevant to the Bangladeshi healthcare context.

Workplace violence was defined according to the WHO as physical, verbal, psychological, or sexual violence occurring in relation to professional duties. Eligible publications reported one or more outcomes related to the prevalence, forms of violence, perpetrators, associated risk factors, organizational or health‐system determinants, consequences, institutional responses, or prevention strategies.

Editorials, letters, conference abstracts, case reports, duplicate publications, and articles lacking sufficient methodological or outcome information were excluded.

### 2.4 Study Selection Process

All retrieved records were imported into reference management software for duplicate removal. Before formal screening, the eligibility criteria were independently piloted by the three authors using a sample of retrieved articles to ensure consistent interpretation. Titles, abstracts, and subsequently full‐text articles were independently screened according to the predefined eligibility criteria. Any differences in study selection were resolved through discussion until consensus was achieved. Agreement among reviewers remained consistently high throughout the screening process; however, formal inter‐reviewer agreement statistics (e.g., Cohen's *κ*) were not calculated because of the relatively small number of included studies.

A total of 32 records were identified through database searching and citation tracking. After duplicate removal, titles and abstracts were screened, followed by full‐text assessment of potentially eligible articles. Ultimately, 12 publications were retained for the structured narrative synthesis, comprising contemporary Bangladeshi primary evidence together with selected contextual and comparative evidence. A PRISMA‐based flow diagram (Figure 1) was used to improve transparency in reporting the literature identification, screening, eligibility assessment, and inclusion process, although PRISMA was originally developed for systematic reviews.

**FIGURE 1:**
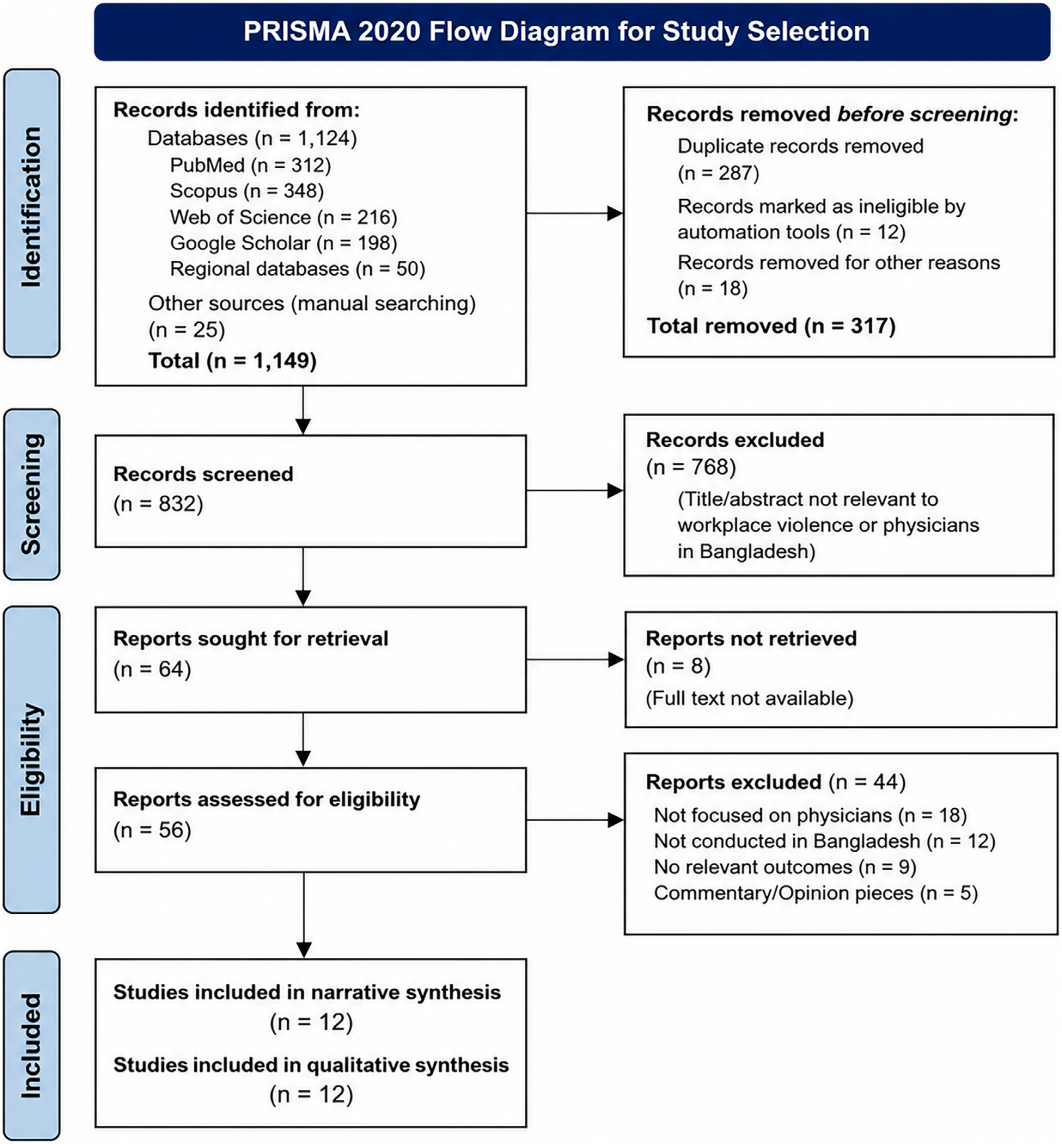
PRISMA‐based study selection flowchart.

### 2.5 Data Extraction

Data were extracted using a standardized extraction form developed specifically for this review and pilot‐tested before full data extraction. Information extracted included publication year, evidence type, study design, country or setting, participant characteristics, sample size, measurement tools, recall period (where applicable), prevalence and types of workplace violence, perpetrators, associated risk factors, organizational and health‐system determinants, reported consequences, institutional responses, prevention strategies, methodological strengths and limitations, and key findings.

Data extraction was performed independently by the three authors, with discrepancies resolved through discussion until consensus was reached. Where study information was incomplete or unclear, findings were interpreted cautiously during evidence synthesis.

### 2.6 Quality Assessment

The methodological quality of primary observational studies was assessed using the appropriate Joanna Briggs Institute (JBI) critical appraisal checklists, primarily the checklist for analytical cross‐sectional studies because most original investigations employed cross‐sectional designs. The appraisal considered inclusion criteria, sampling methods, validity and reliability of outcome measurement, management of confounding factors, statistical analysis, response rates, and completeness of reporting.

Narrative reviews, systematic reviews, content analyses, and expert commentaries were assessed according to methodological transparency, relevance, and contribution to the review rather than using JBI tools designed exclusively for primary research. Quality appraisal was conducted independently by the three authors, and disagreements were resolved through consensus. The appraisal informed interpretation of the evidence, but publications were not excluded solely on the basis of methodological quality. Detailed appraisal results are presented in Table S2.

### 2.7 Data Synthesis

Because substantial heterogeneity existed across study designs, evidence types, participant populations, healthcare settings, and outcome measures, quantitative meta‐analysis was not appropriate. Instead, findings were synthesized narratively using an inductive thematic approach. Themes were generated by comparing study findings and grouping similar concepts across publications before being refined iteratively according to the review objectives. The final synthesis examined patterns of workplace violence, associated risk factors, perpetrators, organizational and health‐system determinants, consequences, institutional responses, and prevention strategies.

Where findings differed across studies, interpretations considered methodological quality, study design, healthcare setting, participant characteristics, and outcome definitions to provide a balanced and contextually appropriate synthesis. The integrated analysis was used to identify consistent evidence, important knowledge gaps, and implications for future research, policy, and practice.

## 3 Results

### 3.1 Study Selection and Characteristics

The contemporary Bangladeshi primary evidence formed the core evidence base, whereas earlier Bangladeshi and international publications were used to contextualize determinants, mechanisms, consequences, and prevention. Their methodological characteristics and principal contributions are summarized in Table 1. The included evidence comprised facility‐based and nationwide cross‐sectional studies, content analyses, narrative reviews, systematic reviews, and contextual evidence from comparable healthcare settings. Most primary studies were conducted in tertiary hospitals and other large urban healthcare facilities in Bangladesh, particularly in Dhaka, and collectively represented more than 6000 healthcare professionals, including physicians.

**TABLE 1:** Characteristics and methodological summary of evidence included in the narrative review.

Author [reference]	Evidence type	Country/Setting	Population/Source	Sample size	Measurement tool/data source	Main contribution to the review	Quality appraisal^a^	Key limitations
**Hasan et al**. [6]	Facility‐based cross‐sectional study	Public tertiary hospitals, Bangladesh	Physicians	468	Structured self‐administered questionnaire	Reported a high prevalence of workplace violence, predominantly psychological violence. Younger physicians, emergency department duty, and inadequate security were associated with increased violence risk	High	Cross‐sectional design; self‐reported data; tertiary hospitals only
**Rony et al**. [7]	Nationwide cross‐sectional study	Public and private healthcare facilities, Bangladesh	Healthcare workers	3977	Standardized structured questionnaire	Identified verbal abuse as the most common form of violence. Patient relatives were the principal perpetrators. Violence was associated with burnout, fear, and reduced job satisfaction	High	Included multiple healthcare professions; physician‐specific estimates limited
**Shahjalal et al**. [8]	Cross‐sectional study	Tertiary hospitals, Bangladesh	Physicians	406	Structured questionnaire	Identified predictors of physical violence, including younger age, male sex, unmarried status, emergency department duty, and night shifts	High	Cross‐sectional design; tertiary hospitals only
**Hasan et al**. [9]	Content analysis	Bangladesh	Nationally reported workplace violence incidents	Not applicable	Content analysis of documented incidents	Demonstrated that psychological violence was more frequent than physical violence and highlighted underreporting and normalization of workplace violence	Moderate	Based on reported incidents; reporting bias likely
**Shahjalal et al**. [10]	Cross‐sectional study	Public and private health facilities, Bangladesh	Healthcare professionals	1081	Structured questionnaire	Demonstrated higher workplace violence in public facilities and identified organizational failures, inadequate staffing, and poor security as important contributing factors	High	Mixed healthcare professions; self‐reported data
**Caruso et al**. [11]	Systematic review	International	Physicians	Multiple studies	Systematic literature review	Synthesized international evidence demonstrating associations between workplace violence, burnout, anxiety, depression, reduced professional performance, and workforce attrition, and summarized institutional prevention strategies	High	Findings not specific to Bangladesh; heterogeneity among included studies
**Phillips** [12]	Narrative review	The United States	Healthcare workers	Not applicable	Literature review	Described patterns, causes, consequences, and institutional responses to workplace violence in healthcare settings, providing international context	Moderate	Focused primarily on the United States; not physician‐specific throughout
**Kader et al**. [13]	Qualitative content analysis	Bangladesh	Reported incidents involving physicians	157 reported incidents	Media and documented incident reports	Identified treatment delays, “power practice,” patient deaths, communication failures, and inadequate infrastructure as major triggers of workplace violence	Moderate	Secondary data source; dependent on publicly reported incidents
**Soron** [14]	Narrative commentary	Bangladesh	Healthcare context	Not applicable	Expert commentary	Discussed psychological and sociocultural mechanisms underlying violence against physicians, including grief reactions, mistrust, unrealistic expectations, and emotional displacement	Moderate	Commentary rather than empirical research
**Mohiuddin** [15]	Narrative literature review	Bangladesh	Healthcare system	Not applicable	Literature review	Examined patient satisfaction, communication barriers, health literacy, and health system limitations contributing to hostility toward healthcare professionals	Moderate	Not specifically designed to evaluate workplace violence
**Shaikh et al**. [16]	Cross‐sectional study	Pakistan	Healthcare workers	>8000	Structured questionnaire	Demonstrated high levels of workplace violence associated with overcrowding, inadequate staffing, and governance deficiencies, providing contextual evidence from a comparable South Asian setting	High	Conducted outside Bangladesh; included multiple healthcare professions
**Chakraborty et al**. [17]	Systematic literature review	Global	Physicians and nurses	Multiple studies	Systematic literature review	Synthesized global evidence on determinants, consequences, and prevention of workplace violence, highlighting the importance of organizational policies, communication, security, and workforce support	High	Global evidence; substantial heterogeneity across included studies

^a^ Quality appraisal was performed using the appropriate Joanna Briggs Institute (JBI) critical appraisal tools for primary studies. Narrative reviews, systematic reviews, and expert commentaries were appraised according to methodological transparency, relevance, and contribution to the narrative synthesis rather than using JBI checklists designed for primary research.

Overall, the methodological quality of the included evidence ranged from moderate to high. Most primary studies employed clearly defined eligibility criteria, appropriate sampling methods, validated measurement approaches, and suitable statistical analyses. Frequently reported methodological limitations included cross‐sectional study designs, reliance on self‐reported data, incomplete adjustment for potential confounding factors, limited reporting of response rates, and restricted generalizability because many studies were conducted in urban tertiary healthcare settings. These methodological considerations informed interpretation of the findings throughout the narrative synthesis. Detailed study characteristics and quality appraisal findings are presented in Table 1 and Table S2, respectively.

### 3.2 Prevalence and Patterns of Workplace Violence

The included studies consistently demonstrated that workplace violence is a common occupational hazard among physicians and healthcare professionals in Bangladesh, although reported prevalence varied according to study population, healthcare setting, recall period, and operational definitions. Rather than presenting the evidence study by study, findings were synthesized according to recurring thematic domains to facilitate comparison of patterns, contextual differences, and areas of uncertainty across the literature. Physician‐specific studies reported a substantial burden of workplace violence, whereas studies involving broader healthcare‐worker populations also reported high levels of exposure. Because population composition, recall periods, and outcome definitions differed across studies, these estimates should not be interpreted as directly comparable physician‐specific prevalence estimates [13, 14, 15].

Across the available evidence, psychological violence, including verbal abuse, threats, intimidation, and bullying, was consistently reported more frequently than physical violence [13, 14, 15, 16]. Physical assaults occurred less frequently but remained an important concern, particularly in emergency departments, tertiary hospitals, and other high‐acuity clinical settings [13, 14, 15]. Several studies also reported a greater frequency of workplace violence in public healthcare facilities than in private institutions, particularly in environments characterized by overcrowding, heavy patient workloads, and limited institutional resources [13, 14, 17].

### 3.3 Demographic and Occupational Risk Factors

Several demographic and occupational characteristics were consistently associated with an increased likelihood of workplace violence (Table 2). Younger physicians, particularly those younger than 30 years, experienced higher rates of violence than more senior physicians [14]. Early‐career clinicians, including interns and resident physicians, were also identified as particularly vulnerable.

**TABLE 2:** Demographic and occupational risk factors associated with workplace violence against physicians.

Risk factor category	High‐risk subgroup	Adjusted odds ratio (AOR)^a^ [95% CI]	Qualitative context
Age	Physicians aged <30 years	2.14 [1.08–4.23]	Early‐career physicians, including interns and residents, were disproportionately affected because of frequent frontline exposure and limited professional experience [14]
Marital status	Never‐married physicians	3.02 [1.45–6.31]	Elevated risk may reflect younger age, lower workplace seniority, and reduced institutional authority [14]
Hospital type	Public hospitals	2.74 [1.99–3.76]	Public hospitals experienced substantially higher violence exposure because of overcrowding, workforce shortages, resource constraints, and inadequate security systems [17]
Department	Emergency department	2.30 [1.03–5.12]	Emergency and surgery‐related departments demonstrated significantly higher violence exposure because of acute crises and emotionally charged clinical situations [13, 14]
Gender	Male physicians	2.76 [1.23–6.19]	Male physicians were more vulnerable to physical violence, whereas female physicians more commonly experienced verbal and sexual harassment [4, 14]
Shift work	Night‐shift duties	1.52 [1.10–2.11]	Violence incidents were more common during night shifts because of lower staffing levels, reduced supervision, and emergency‐related stress [14]

^a^ Adjusted odds ratios are estimates reported by individual primary studies and were not pooled across studies. Differences in study populations, statistical models, reference categories, outcome definitions, and adjustment variables should therefore be considered when interpreting these estimates.

Gender‐related differences were observed across the included studies. Male physicians more frequently experienced physical violence, whereas female physicians more commonly reported verbal abuse and sexual harassment [14]. Occupational characteristics also influenced exposure. Physicians working in emergency departments, surgical services, and other frontline clinical settings experienced higher levels of workplace violence than those in other specialties [13, 14]. Similarly, night‐duty physicians were reported to be at increased risk, particularly in settings characterized by reduced staffing, limited security, and high patient volumes [14].

### 3.4 Perpetrators and Contributing Factors

Patient relatives and attendants were consistently identified as the principal perpetrators of workplace violence, followed by patients and hospital visitors [8, 15]. Violent incidents frequently involved multiple individuals, with mob participation often increasing the severity of assaults and damage to hospital property [8]. Several studies also reported that perpetrators commonly left healthcare facilities immediately after incidents, creating barriers to identification, reporting, investigation, and legal accountability [8, 15].

Violence most frequently occurred during emotionally stressful clinical situations, including treatment delays, emergency care, deterioration of critically ill patients, and communication of unfavorable clinical outcomes. In one content‐analysis study, treatment delays (26.8%) and “power practice” (26.1%) were among the most frequently reported triggers of violent incidents [8]. Additional reported triggers included patient deaths, perceived treatment failure, inadequate communication, overcrowding, insufficient security measures, limited institutional resources, and weak patient management systems [8, 9, 10, 15, 17].

To facilitate evidence synthesis, the identified determinants were organized according to the social ecological model (Table 3 and Figure 2). At the individual level, younger age, early‐career status, and night‐duty work were associated with greater vulnerability. Interpersonal determinants included aggressive behavior by patient relatives, unrealistic treatment expectations, grief following patient death, and ineffective communication. Institutional determinants encompassed inadequate staffing, overcrowding, prolonged waiting times, insufficient security, and weak reporting mechanisms, whereas broader health‐system and societal determinants included physician shortages, limited infrastructure, governance challenges, low health literacy, financial hardship, negative media portrayal, and political influence. The significance of these exposures becomes clearer when their consequences for physicians, healthcare organizations, and healthcare delivery are considered.

**TABLE 3:** Multilevel determinants of workplace violence against physicians in Bangladesh based on the social ecological model.

Social ecological level	Determinants identified in included studies	Representative evidence
**Individual**	Younger age (<30 years), early‐career physicians, limited conflict management experience, and night‐duty physicians	[13, 14]
**Interpersonal**	Aggressive patient relatives, unrealistic expectations, grief after patient death, poor physician–patient communication, and treatment dissatisfaction	[8, 9, 10, 15]
**Institutional/Workplace**	Overcrowding, inadequate staffing, prolonged waiting time, insufficient security, weak reporting systems, and inadequate administrative support	[10, 13, 14, 15, 16, 17]
**Health system**	Physician shortages, limited emergency‐care capacity, inadequate infrastructure, weak governance, insufficient legal protection, and limited resources	[11, 12, 15, 17]
**Societal**	Low health literacy, negative media portrayal, political influence (‘power practice’), financial hardship, and declining public trust in healthcare	[8, 9, 10]

Abbreviation: SEM, social ecological model.

**FIGURE 2:**
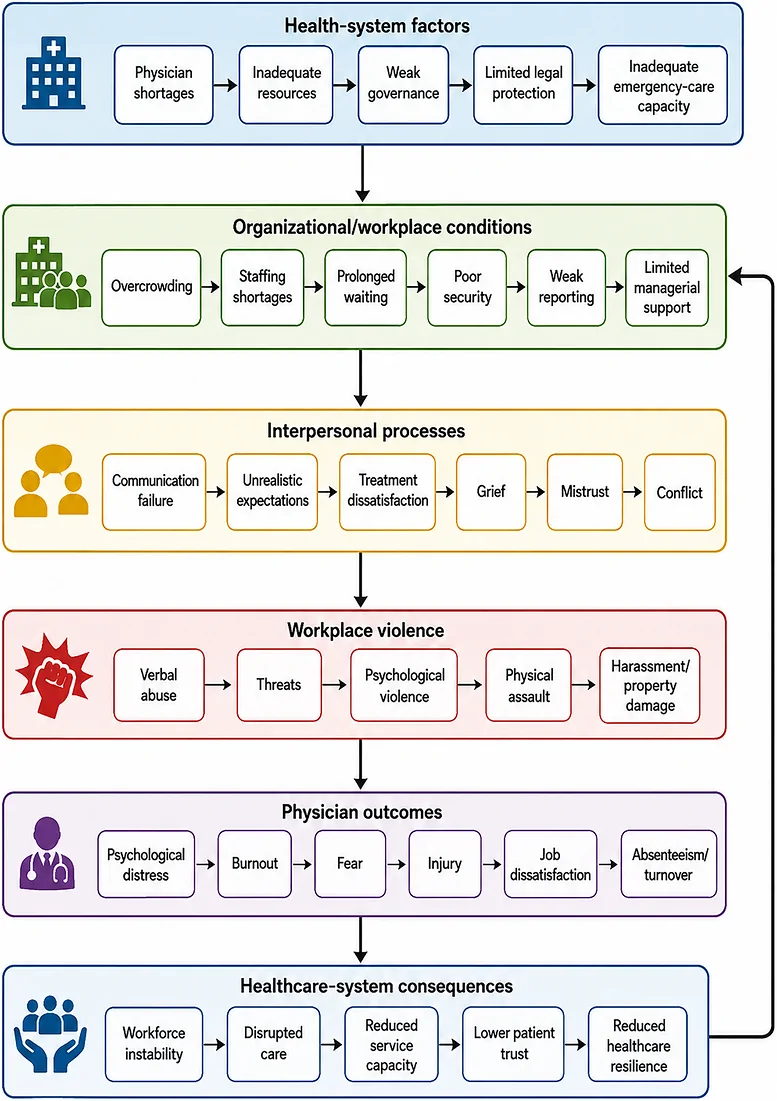
Integrated socio‐ecological conceptual framework illustrating pathways from health‐system and organizational conditions to workplace violence against physicians and subsequent physician and healthcare‐system consequences.

### 3.5 Consequences of Workplace Violence

The reviewed evidence consistently demonstrated that workplace violence was associated with important physical, psychological, professional, organizational, and health‐system consequences. Physicians exposed to violent incidents frequently reported psychological distress, anxiety, depression, emotional trauma, fear, burnout, compassion fatigue, and symptoms consistent with post‐traumatic stress disorder [4, 13, 15]. Severe physical assaults occasionally resulted in injuries requiring medical attention [13, 14].

Occupational consequences included reduced job satisfaction, diminished professional motivation, defensive medical practice, absenteeism, and intentions to leave clinical practice or migrate to safer working environments [4, 15]. Several publications also described broader impacts on healthcare delivery, including disruption of clinical services, deterioration of physician–patient relationships, increased institutional burden, and reduced willingness of physicians to work in high‐risk specialties, such as emergency medicine and critical care [4, 6, 7].

Building on the multilevel determinant framework presented in Table 3 and the multidimensional consequences summarized in Table 4, the evidence was further synthesized across broader thematic domains to identify recurring patterns, areas of convergence and contextual variation, and their implications for prevention. These cross‐cutting patterns are summarized in Table 5. Identifying the determinants and consequences of violence is insufficient, however, unless healthcare institutions possess effective mechanisms to prevent, report, investigate, and respond to violent incidents.

**TABLE 4:** Multidimensional consequences of workplace violence against physicians.

Impact domain	Reported consequences	Evidence
**Physical**	Physical injuries requiring medical treatment, bruises, fractures, soft tissue injuries, and reduced physical safety	[13, 14]
**Psychological**	Anxiety, depression, emotional trauma, fear, burnout, compassion fatigue, and post‐traumatic stress symptoms	[4, 13, 15]
**Professional**	Reduced job satisfaction, defensive medical practice, absenteeism, decreased motivation, and intention to leave clinical practice	[4, 15]
**Organizational**	Reduced workforce morale, staff turnover, disruption of healthcare delivery, weakened teamwork, and increased administrative burden	[4, 6, 7]
**Health system**	Workforce shortages, reduced service quality, deterioration of physician–patient relationships, and reduced willingness to work in emergency and critical care settings	[4, 6, 7, 11, 12]
**Economic**	Productivity losses, staff replacement costs, increased security expenditures, legal costs, and reduced institutional efficiency^a^	[4]

^a^ Economic consequences were primarily derived from international evidence because direct economic evaluations from Bangladesh remain limited.

**TABLE 5:** Thematic synthesis of workplace violence against physicians in Bangladesh: convergent evidence, contextual variation, and implications.

Thematic domain	Convergent evidence	Interpretation	Evidence consistency^a^	Implications
**Individual/Occupational**	Younger and early‐career physicians; emergency and night‐duty staff	Differential exposure to high‐risk clinical environments may increase exposure to violent encounters	Relatively consistent	Targeted orientation, supervision, occupational‐safety, and de‐escalation training
**Interpersonal**	Relatives/Attendants; communication failures; treatment dissatisfaction; and grief following adverse outcomes	Violence may emerge during emotionally charged encounters involving unmet expectations, distress, and communication breakdown	Consistent but context‐dependent	Communication, expectation management, conflict resolution, and de‐escalation
**Organizational/Workplace**	Overcrowding, prolonged waiting, staffing shortages, inadequate security, and weak reporting	Work organization and institutional preparedness may create conditions that facilitate escalation and reduce effective response	Relatively consistent	Staffing, waiting‐time management, security, reporting, and institutional response systems
**Health system**	Physician shortages, limited infrastructure, emergency‐care constraints, and governance weaknesses	Structural health‐system pressures may intensify organizational strain and indirectly increase exposure to violence	Consistent but context‐dependent	Workforce planning, infrastructure strengthening, governance, and accountability
**Societal**	Low health literacy, unrealistic expectations, negative media portrayal, financial hardship, and political influence	Broader social and governance conditions shape trust, expectations, conflict, and institutional accountability	Variable across studies	Public engagement, health literacy, responsible communication, and governance reform
**Physician and healthcare consequences**	Psychological distress, burnout, fear, injury, reduced job satisfaction, absenteeism, turnover, and disrupted care	Violence may undermine physician well‐being, workforce stability, and continuity of healthcare delivery	Relatively consistent	Psychological support, occupational rehabilitation, workforce retention, and resilience measures
**Institutional prevention and response**	Weak reporting, limited investigation, inadequate security, and insufficient managerial/legal support	Weak institutional responses may reinforce underreporting and normalization and limit organizational learning	Consistent but incompletely evaluated	Standardized reporting, investigation, rapid response, accountability, and post‐incident support

^a^ Evidence consistency reflects the degree to which similar patterns recurred across studies and contexts and does not represent a formal certainty‐of‐evidence grading.

### 3.6 Reporting Practices and Institutional Responses

Institutional responses to workplace violence were consistently reported to be inadequate across many healthcare settings in Bangladesh. Several studies found that substantial proportions of violent incidents were not formally investigated and that many perpetrators experienced no administrative or legal consequences following reported events [15, 17]. Underreporting was commonly attributed to limited confidence in reporting mechanisms, fear of retaliation, lack of administrative support, and the perception that workplace violence was an unavoidable aspect of medical practice [15, 16].

The reviewed evidence further indicated that many healthcare institutions lacked standardized incident reporting systems, comprehensive workplace violence prevention policies, adequate security infrastructure, and structured psychological support services for affected physicians [15, 17]. Several publications also highlighted that normalization of workplace violence within healthcare organizations discouraged reporting and weakened institutional accountability, thereby limiting opportunities for prevention, organizational learning, and continuous quality improvement. Taken together, the evidence can be organized into interconnected thematic domains spanning individual and occupational vulnerability, interpersonal processes, organizational and health‐system conditions, societal influences, consequences for physicians and healthcare delivery, and institutional prevention and response; these cross‐cutting patterns, areas of convergence, and contextual uncertainties are summarized in Table 5.

### 3.7 International Contextual Comparison

The Bangladeshi evidence showed several patterns that were broadly consistent with findings from other South Asian and resource‐constrained healthcare systems. Across India, Pakistan, Nepal, and China, verbal or psychological violence was commonly reported, with patients and their relatives or attendants frequently identified as perpetrators. Recurring contextual determinants included high workload, overcrowding, treatment‐related dissatisfaction, communication difficulties, inadequate staffing or resources, and insufficient institutional security [4, 6, 7]. However, the Bangladeshi literature additionally emphasized physician shortages, limited emergency‐care capacity, governance weaknesses, and inadequate institutional response mechanisms as important structural vulnerabilities [11, 12]. A contextual comparison of these patterns is presented in Table 6. These comparisons should be interpreted cautiously because the underlying studies differed substantially in populations, settings, definitions of workplace violence, recall periods, and measurement approaches.

**TABLE 6:** Contextual comparison of workplace violence against physicians in Bangladesh and selected South Asian and other LMIC settings.

Setting	Common forms/perpetrators	Recurring determinants	Contextual/System characteristics	Prevention emphasis
**Bangladesh**	Verbal and psychological violence; threats and physical violence; patient relatives/attendants frequently implicated [4, 8, 13, 14, 15, 16, 17]	Overcrowding, treatment delays, staffing shortages, poor communication, inadequate security, weak reporting, and governance constraints	Physician shortages, limited emergency‐care capacity, constrained institutional resources, and governance challenges [11, 12]	Governance and accountability, workforce strengthening, reporting systems, security, legal protection, communication, and occupational support
**India**	Verbal and physical violence; patients, relatives/attendants, and other actors may be involved [6, 7]	Workload, treatment‐related dissatisfaction, communication problems, organizational pressures, and inadequate security	Large and heterogeneous healthcare system; government facilities may face substantial workload and security pressures	Multilevel organizational, security, legal, communication, and policy interventions [6, 7]
**Pakistan**	Verbal violence predominates; patients and attendants are frequent perpetrators; physical violence is also reported [6]	Overcrowding, limited resources, inadequate security, workload, and communication‐related factors	Resource and governance constraints and substantial pressure on healthcare facilities	Institutional protection, communication training, security measures, reporting, and policy enforcement [6]
**Nepal**	Verbal and physical violence reported against healthcare professionals [7]	Workload, communication difficulties, organizational pressures, and inadequate security	Resource‐constrained healthcare environment	Institutional prevention, security, reporting, and policy measures [7]
**China**	Verbal violence and threats are prominent; patients and family members/relatives are major perpetrators [4, 7]	Workload, high‐demand clinical settings, doctor–patient interactions, unmet expectations, and organizational factors	Distinct healthcare and regulatory context, with substantial variation across healthcare settings [7]	Structural and organizational measures, prevention/reporting systems, policy, and workforce protection
**Other LMIC settings**	Forms and perpetrators vary across settings	Staffing shortages, workload, overcrowding, inadequate resources, and security, communication problems	Highly heterogeneous health‐system and institutional environments	Multilevel occupational‐health, organizational, governance, and policy interventions [3, 4]

*Note:* Comparisons are contextual rather than quantitative because the underlying studies differed in population, healthcare setting, outcome definitions, recall periods, sampling approaches, and measurement instruments. The table therefore identifies recurring patterns rather than implying direct equivalence in prevalence or effect size across countries.

## 4 Discussion

This review synthesizes the available evidence and indicates that workplace violence against physicians in Bangladesh is not merely an occupational safety issue but also a manifestation of broader structural weaknesses within the healthcare system. The predominance of psychological violence, frequent involvement of patients’ relatives and attendants, and concentration of incidents in tertiary care hospitals and emergency departments indicate that organizational deficiencies, resource constraints, and strained patient–provider relationships are important contextual factors associated with violence. These findings support the view that workplace violence is a complex systems problem shaped by interactions among individual, interpersonal, organizational, healthcare‐system, and societal factors rather than isolated interpersonal conflicts.

The findings are consistent with international evidence showing that psychological violence is a common form of workplace violence experienced by healthcare professionals and may remain underrecognized despite its effects on mental health and professional functioning [1, 3, 4]. Persistent exposure to psychological abuse may contribute to emotional distress, burnout, reduced job satisfaction, diminished professional confidence, and impaired quality of care [3, 4]. Although the included studies consistently identified workplace violence as an important occupational problem, reported prevalence and associations varied across studies. The strongest convergence concerned the predominance of psychological and verbal violence, frequent involvement of patients’ relatives or attendants, and concentration of incidents in high‐pressure clinical environments. In contrast, the magnitude of reported prevalence and the strength of associations with individual demographic characteristics varied according to population, healthcare setting, recall period, outcome definition, sampling, and measurement approach.

The international evidence suggests that workplace violence against physicians in Bangladesh represents part of a broader pattern observed across South Asian and other resource‐constrained healthcare systems, rather than an isolated national phenomenon [4, 6, 7]. The recurring prominence of verbal violence, involvement of patients and relatives, and association with workload, overcrowding, communication difficulties, and inadequate security point to common organizational and service‐delivery pressures across these settings. However, the Bangladeshi context appears to involve additional structural vulnerabilities, particularly physician shortages, limited emergency‐care capacity, constrained institutional resources, and weaknesses in governance and accountability [11, 12]. These differences suggest that interventions cannot simply be transferred from other settings without adaptation to the underlying health‐system context. In Bangladesh, measures focused solely on hospital security or individual communication skills are unlikely to be sufficient unless accompanied by improvements in workforce capacity, emergency‐care organization, institutional reporting, governance, and legal accountability.

Younger physicians, early‐career clinicians, emergency department staff, and those working night shifts were frequently identified as particularly vulnerable. These findings align with the social ecological model, which recognizes workplace violence as the product of interactions between individual vulnerability, interpersonal relationships, organizational conditions, institutional governance, and broader societal influences. A recent Indian narrative review similarly applied a socio‐ecological framework and identified interacting individual, interpersonal, organizational, and structural determinants of workplace violence [18]. Frontline physicians often manage critically ill patients under conditions of heavy workload, diagnostic uncertainty, treatment delays, and limited institutional support, creating circumstances in which emotionally distressed patients or attendants may become aggressive [13, 14]. Importantly, these levels should not be interpreted as independent causes. Rather, individual and occupational characteristics may indicate differential exposure to high‐risk encounters, whereas interpersonal conflict is shaped by organizational conditions and broader health‐system constraints. The SEM therefore provides a mechanism‐oriented interpretation in which workplace violence emerges from the interaction of vulnerabilities and environmental pressures rather than from a single determinant.

The evidence therefore converges more strongly at the level of broad mechanisms than at the level of individual demographic associations. Recurring evidence supports the importance of verbal and psychological violence, relatives or attendants as frequent perpetrators, high‐pressure clinical settings, treatment delays, overcrowding, staffing constraints, communication problems, and inadequate security. Conversely, findings concerning demographic characteristics, such as sex and marital status, are less consistently established and should therefore be interpreted as context‐dependent associations rather than universal risk factors. This distinction is important because differences in sampling, healthcare settings, measurement approaches, and study populations may produce apparently divergent findings even when broader underlying mechanisms are similar.

This review also highlights the important role of sociocultural and governance‐related factors. Violence frequently followed treatment delays, patient deterioration, or unfavorable clinical outcomes, when grief, frustration, and perceived treatment failure were redirected toward physicians. Similar patterns have been reported across South Asian healthcare settings [6, 7, 8]. Limited health literacy, unrealistic expectations regarding medical care, and poor understanding of disease complexity may reinforce perceptions that adverse outcomes primarily reflect physician negligence rather than healthcare‐system limitations [9, 10]. In addition, media coverage emphasizing adverse clinical outcomes without adequate contextual discussion may further erode public trust in healthcare professionals.

The phenomenon of “power practice” further illustrates how political influence, social hierarchy, and weak institutional accountability may facilitate violence while limiting administrative or legal consequences for perpetrators [8]. Comparable governance‐related challenges have been documented in Pakistan, India, and several African countries [4, 7], indicating that effective prevention requires not only improved hospital security but also stronger governance, transparent institutional policies, and consistent legal protection for healthcare workers. This interpretation is consistent with the broader occupational‐health perspective that workplace violence is influenced by work organization, psychosocial working conditions, risk exposure, institutional preparedness, and organizational responses.

The WHO–ILO framework for addressing workplace violence in the health sector emphasizes prevention, risk assessment, incident management, worker care and support, and monitoring, which correspond closely to the organizational and health‐system deficiencies repeatedly identified in the Bangladeshi evidence [2]. The Occupational Safety and Health Administration (OSHA) similarly emphasizes a comprehensive workplace violence prevention approach incorporating hazard assessment, institutional policies, reporting and investigation procedures, security measures, training, recordkeeping, and support for affected workers. Although OSHA guidance reflects the US occupational‐safety context and does not constitute Bangladeshi regulation, these principles provide a useful international benchmark for developing facility‐level prevention and response systems.

Institutional responses to workplace violence remain inadequate across many healthcare settings in Bangladesh. Underreporting is common because of limited confidence in reporting systems, fear of retaliation, and the normalization of violence as an unavoidable aspect of medical practice [15, 16, 17]. Inadequate post‐incident support, including psychological counseling, legal assistance, and occupational rehabilitation, may further exacerbate burnout, workforce attrition, and reduced organizational resilience. These findings reinforce the importance of treating reporting and institutional response as integral components of prevention rather than merely administrative procedures.

The consequences of workplace violence extend well beyond individual physicians. Violence may adversely affect clinical decision‐making, communication, teamwork, and professional confidence while contributing to burnout, absenteeism, defensive medical practice, and intentions to leave the profession [3, 4]. In a resource‐constrained healthcare system, such as Bangladesh, these effects may further worsen workforce shortages and compromise healthcare quality and accessibility [11, 12]. Although workplace violence is also likely to impose economic costs through productivity losses, staff turnover, legal proceedings, and increased security expenditures, these impacts remain insufficiently investigated.

Several emerging issues warrant further attention. Although most included studies were conducted after the COVID‐19 pandemic, its longer term effects on workforce shortages, accumulated service backlogs, and institutional strain may continue to influence workplace violence. Similarly, digital harassment through social media, gender‐specific experiences of violence, and differences between urban and rural healthcare settings remain underexplored and represent important priorities for future research.

Overall, the thematic synthesis indicates that preventing workplace violence requires coordinated action across multiple levels. Healthcare institutions should strengthen reporting systems, security infrastructure, rapid‐response mechanisms, psychological support services, and zero‐tolerance policies. National efforts should reinforce legal protection for healthcare workers through effective implementation and institutional accountability, whereas undergraduate and postgraduate medical education should incorporate communication skills, conflict resolution, violence de‐escalation, and occupational safety training. Public engagement initiatives aimed at improving health literacy, fostering realistic expectations of healthcare, and strengthening trust between healthcare providers and communities are equally important. A comprehensive systems‐based approach addressing organizational, legal, educational, and societal determinants is therefore essential for creating safer healthcare environments and strengthening the long‐term resilience and sustainability of Bangladesh's healthcare workforce.

### 4.1 Limitations, Implications, and Future Research

Several limitations should be considered when interpreting the findings of this review. Most included studies employed cross‐sectional designs, precluding causal inference. Selection, recall, reporting, and social desirability biases may have influenced the findings because many studies relied on voluntary participation and self‐reported experiences. Restricting the review to English‐language publications may have introduced language bias, whereas publication bias remains possible because studies describing severe or widely publicized incidents are more likely to be published. Furthermore, the narrative synthesis involved interpretative judgment, and substantial heterogeneity in study designs, participant characteristics, healthcare settings, recall periods, and outcome definitions precluded quantitative meta‐analysis. Finally, because most evidence originated from urban tertiary hospitals, the findings may not be fully generalizable to rural or primary healthcare settings.

These limitations reduce confidence in the precision of individual prevalence estimates and in causal interpretations, but they do not necessarily negate the broader qualitative patterns identified across multiple studies. Greater confidence can be placed in recurring themes observed across different settings, such as the prominence of verbal and psychological violence, frequent involvement of patient relatives or attendants, and the contribution of organizational pressures. In contrast, conclusions regarding the magnitude of specific demographic associations and causal relationships should be interpreted more cautiously because they are particularly susceptible to differences in sampling, measurement, recall period, and study design.

Despite these limitations, this review provides a comprehensive synthesis of contemporary evidence on workplace violence against physicians in Bangladesh and identifies important gaps in healthcare governance, institutional safety, workforce protection, and system preparedness. The findings support the implementation of standardized reporting systems, rapid‐response security protocols, structured psychological support services, mandatory incident documentation, and occupational health programs. Strengthening communication skills, conflict management, violence de‐escalation, and occupational safety training within medical education, together with effective enforcement of legislation protecting healthcare workers, should be key policy priorities. These measures should be integrated into broader healthcare governance, workforce planning, quality‐improvement, and health‐system‐resilience strategies rather than implemented solely as security responses.

Future research should prioritize prospective longitudinal and intervention studies to better understand the long‐term consequences of workplace violence and evaluate the effectiveness of prevention strategies, reporting systems, security measures, and institutional policies. Greater emphasis should also be placed on qualitative and mixed‐methods research exploring the perspectives of physicians, patients, caregivers, hospital administrators, and policymakers, as well as investigations of rural and primary healthcare settings, gender‐specific experiences, digital harassment, and the economic burden of workplace violence. Comparative studies across public and private facilities, urban and rural settings, and primary, secondary, and tertiary levels of care would further clarify how organizational and health‐system contexts influence violence and the effectiveness of preventive strategies. A coordinated research agenda for Bangladesh and similar resource‐constrained healthcare systems should therefore prioritize four interconnected areas: (1) development of standardized operational definitions, validated measurement instruments, and nationwide surveillance mechanisms to enable meaningful comparison across settings and over time; (2) longitudinal and mixed‐methods research examining the temporal, organizational, interpersonal, and health‐system pathways through which workplace violence develops and affects physicians and healthcare delivery; (3) rigorous evaluation of organizational, security, communication, reporting, psychological‐support, governance, and policy interventions, including their feasibility, effectiveness, sustainability, and economic implications; and (4) comparative research across healthcare settings and health systems to identify context‐specific and transferable prevention strategies. Such an agenda would strengthen the evidence base for Bangladesh while providing a potentially transferable framework for other resource‐constrained health systems experiencing similar occupational safety and workforce‐resilience challenges.

## 5 Conclusion

This narrative review demonstrates that workplace violence against physicians in Bangladesh is not simply an interpersonal or security problem but a systems‐level occupational, organizational, and public health challenge. The available evidence suggests that violence emerges through interactions among physician vulnerability, emotionally charged patient encounters, organizational pressures, health‐system constraints, governance weaknesses, and broader sociocultural influences. Although methodological heterogeneity limits precise estimation and causal interpretation, recurring patterns across the evidence identify several modifiable points for prevention.

Effective prevention therefore requires more than reactive security measures. Healthcare institutions should strengthen risk assessment, staffing, communication, reporting, investigation, security, and post‐incident support, whereas policymakers should address workforce capacity, governance, legal protection, and accountability. Future research should establish standardized measurement, expand evidence beyond tertiary urban hospitals, evaluate interventions prospectively, and examine the effects of workplace violence on physician retention, patient trust, quality of care, and health‐system resilience. Protecting physicians should consequently be regarded as an essential component of building a safe, resilient, and sustainable healthcare system in Bangladesh. Workplace violence should therefore be incorporated into occupational safety, healthcare governance, workforce planning, quality improvement, and health‐system‐resilience agendas rather than managed solely as a hospital security issue.

## Author Contributions

**Md. Abu Sufian**: conceptualization, data curation, formal analysis, investigation, methodology, visualization, writing – original draft, and writing – review and editing. **Nawshin Sayara Satter**: investigation, methodology, quality assessment, validation, writing – review and editing. **Md. Monir Hossain Shimul**: conceptualization, data curation, formal analysis, methodology, supervision, validation, visualization, writing – original draft, and writing – review and editing.

## Funding

The authors have nothing to report.

## Ethics Statement

Ethical approval was not required for this review because the study synthesized evidence from previously published literature and did not involve direct participation of human subjects or collection of primary data. All included studies were reviewed and cited appropriately to maintain academic integrity and transparency.

## Consent

The authors have nothing to report.

## Conflicts of Interest

The authors declare no conflicts of interest.

## Supporting information




**Supporting Table 1:** Complete database search strategy used for the narrative review.
**Supporting Table 2:** Quality appraisal of included studies using the Joanna Briggs Institute (JBI) critical appraisal checklist.

## Data Availability

The data supporting the conclusions of this article are included within the article.
