# Supplementary material for: Workplace Violence Against Physicians in Bangladesh: A Contemporary Systems‐Based Narrative Review of Current Evidence
Source: Public Health Chall. 2026 Nov 7;5(4):e70452. doi: 10.1002/puh2.70452 (PMC13744623; doi:10.1002/puh2.70452)
Supplement: Supplementary file 1 [file PUH2-5-e70452-s001.docx]

**Supplementary Tables**

**Supplementary Table S1. Complete Database Search Strategy Used for the Narrative Review**

| **Database** | **Search Strategy** | **Filters/Limitations Applied** | **Records Retrieved** |
| --- | --- | --- | --- |
| **PubMed** | (“workplace violence” OR violence OR assault OR aggression OR bullying) AND (physician OR doctor OR “healthcare worker”) AND (Bangladesh OR “Bangladeshi healthcare settings”) | English language; Publication years 2022–May 2026; Human studies | n = 10 |
| **Scopus** | TITLE-ABS-KEY (“workplace violence” OR assault OR aggression) AND TITLE-ABS-KEY (physician OR doctor OR “healthcare worker”) AND TITLE-ABS-KEY (Bangladesh) | English language; Article type; Publication years 2022–May 2026 | n = 8 |
| **Web of Science** | TS = (“workplace violence” OR violence OR assault) AND TS = (physician OR doctor OR “healthcare worker”) AND TS = (Bangladesh) | English language; Publication years 2022–May 2026 | n = 6 |
| **Google Scholar** | “violence against physicians in Bangladesh” OR “workplace violence against doctors in Bangladesh” | Publication years 2022–May 2026; First 100 relevant results screened | n = 8 |
| **Manual reference screening** | Reference lists of included articles and related review studies were manually screened to identify additional eligible studies | Studies relevant to workplace violence against physicians in Bangladesh | n = 4 |
| **Regional sources/manual search** | Additional contextual reports and regional publications related to healthcare violence in Bangladesh were reviewed | English language | n = 2 |

**Supplementary Table S2. Quality Appraisal of Included Studies Using the Joanna Briggs Institute (JBI) Critical Appraisal Checklist**

| **Study** | **Appropriate Sampling Strategy** | **Valid and Reliable Measurement** | **Confounding Factors Identified** | **Appropriate Statistical Analysis** | **Response Rate Adequate** | **Overall Methodological Quality** |
| --- | --- | --- | --- | --- | --- | --- |
| Hasan MJ et al. [13] | Yes | Yes | Partial | Yes | Yes | Moderate to High |
| Shahjalal M et al. [14] | Yes | Yes | Yes | Yes | Yes | High |
| Rony MKK et al. [15] | Yes | Yes | Partial | Yes | Yes | Moderate to High |
| Bulbul MMI et al. [16] | Partial | Yes | No | Yes | Partial | Moderate |
| Islam SS et al. [17] | Yes | Yes | Partial | Yes | Yes | Moderate to High |
| Byon HD et al. [8] | Yes | Yes | Not applicable | Yes | Not applicable | Moderate |
| Shaikh S et al. [6] | Yes | Yes | Yes | Yes | Yes | High |
| Caruso R et al. [4] | Yes | Yes | Yes | Yes | Not applicable | High |
| Chakraborty S et al. [7] | Yes | Yes | Yes | Yes | Not applicable | High |
| Soron TR [9] | Partial | Partial | No | Partial | Not applicable | Low to Moderate |
| Mohiuddin AK [10] | Partial | Partial | No | Partial | Not applicable | Low to Moderate |
